# Vascular Anomalies in Pancreaticoduodenectomy: A Lesson Learned

**DOI:** 10.1155/2016/5792980

**Published:** 2016-04-21

**Authors:** Joana E. Ochoa, David T. Pointer, John B. Hamner

**Affiliations:** Department of Surgery, Tulane University School of Medicine, New Orleans, LA 70112, USA

## Abstract

It is essential to identify any variant anatomy prior to surgery as this could have a drastic effect on surgical planning. We describe a case in which two vascular irregularities, an Arc of Buhler and celiac stenosis, were identified on angiogram after completion of a pancreaticoduodenectomy. While there could have been catastrophic results from his surgery in the setting of celiac stenosis, the presence of the aberrant Arc of Buhler allowed this patient to emerge without any permanent morbidity.

## 1. Introduction

The gastrointestinal system is supplied mainly by three vessels, the celiac trunk and the superior and inferior mesenteric arteries. There are numerous anatomical variants that exist between these arteries, many of which regress during embryological development. The embryological remnants of arterial communications between the celiac trunk and the superior mesenteric artery (SMA) collectively are derived from the persistence of the Tandler longitudinal anterior primitive anastomosis [1]. The Arc of Buhler (AOB), specifically, is a direct connection between the celiac trunk and SMA that travels vertically, ventral to the aorta. First described in 1904 by Buhler [2], the anomaly is thought to be a persistence of embryologic circulation, a remnant of the 10th and 13th primitive arteries, comprising the ventral anastomosis [2–5]. It is thought to have an incidence rate of 1–4% [4–7]. It is usually asymptomatic and found incidentally after evaluation for other pathologies [4, 6].

Median arcuate ligament syndrome (MALS) is defined as postprandial abdominal pain, nausea/vomiting, and weight loss due to celiac artery compression from the median arcuate ligament and surrounding structures [8]. Normally the median arcuate ligament passes anteriorly around the aorta above the level of the celiac axis, but in a portion of the population the median arcuate ligament passes anterior to the celiac axis causing compression [8]. There are reports of increasing diagnosis of this syndrome with a wide range of incidence described in the literature from 4 to 24% [9]. Symptoms for MALS are also variable given varying degrees of obstruction and it is often asymptomatic. We describe a patient who was incidentally found to have an AOB in the presence of celiac stenosis due to MALS after he presented with a gastrointestinal bleed following a pancreaticoduodenectomy (Whipple procedure) for a pancreatic mass.

## 2. Case Summary

**Figure 1:**
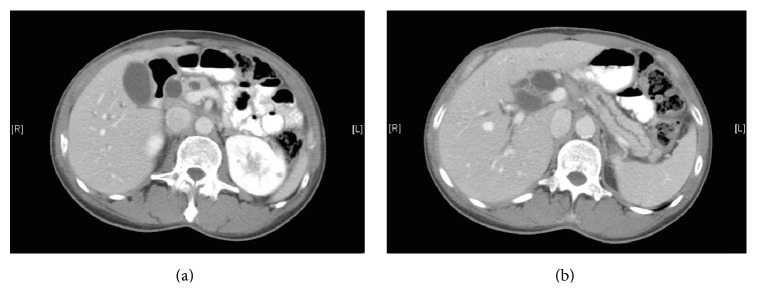
(a) CT abdomen with contrast demonstrating intra- and extrahepatic biliary ductal dilatation. (b) CT demonstrating obvious pancreatic ductal dilatation without identification of discrete mass.

A 58-year-old male with no significant medical or surgical history presented to the emergency department after his coworkers noticed scleral icterus. A 1-week history of darkened urine, vague abdominal pain, and acholic stools was noted upon further questioning. He reported a smoking history of 15 pack-years and substantial daily alcohol intake, though he reported no family history significant for malignant disease. Laboratory values on presentation demonstrated total bilirubin of 15.5 mg/dL, direct bilirubin of 11.7 mg/dL, and alkaline phosphatase of 951 U/L. Subsequent abdominal ultrasound revealed significant intra- and extrahepatic bile duct dilatation (CBD 2.0 cm) with concomitant pancreatic ductal dilatation. CT abdomen and pelvis demonstrated similar findings, without identifying a culprit mass (Figure 1).

An endoscopic retrograde cholangiopancreatography (ERCP) and endoscopic ultrasound (EUS) were performed the following day confirming ductal dilatation and identifying 32.5 mm by 31.8 mm ill-defined periampullary hypoechoic mass. EUS-guided fine needle aspiration was performed resulting in a diagnosis of pancreatic adenocarcinoma. There was no evidence of celiac lymphadenopathy or distant metastatic disease. The portal vein, superior mesenteric artery, and celiac axis were not involved with the lesion. Surgical oncology was consulted for resection. His CA 19-9 was found to be 793. Given findings on initial imaging and EUS/ERCP, the tumor appeared resectable and no further radiological studies were deemed necessary. At this time, after review by radiologists and surgeons, no other abnormalities were noted on the radiologic studies. After discussing treatment options with the patient and his family, the decision was to proceed with surgical resection.

The patient was taken to the operating room where an initial diagnostic laparoscopy demonstrated no metastatic disease. An open pancreaticoduodenectomy was then performed with no complications. There was no noticeable vascular anomaly or aberrant anatomy during the case. Pathologically, the tumor measured 7 × 3 × 2.8 cm identified as moderately differentiated pancreatic ductal adenocarcinoma with 23 lymph nodes negative for malignancy. The tumor was staged at T3N0M0 (Stage IIA) per the American Joint Committee on Cancer guidelines [10].

**Figure 2:**
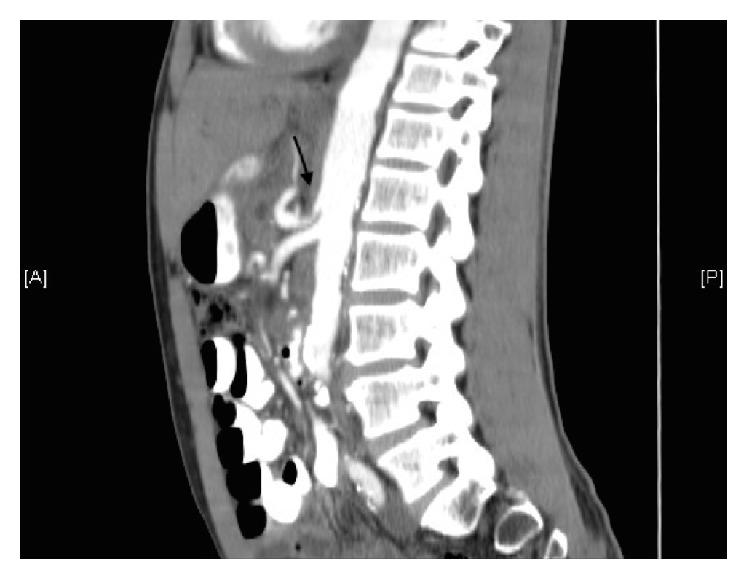
CT abdomen and pelvis sagittal view demonstrating severe stenosis at the origin of the celiac artery.

On postoperative day five, the patient experienced a significant decrease in his hematocrit level from 32.5 to 26.7% along with complaints of increased abdominal pain. After a poor transfusion response, a CT angiography was performed and revealed an intraluminal blush in the small bowel likely at the hepaticojejunostomy site. High-grade stenosis of the celiac artery was also demonstrated (Figure 2).

**Figure 3:**
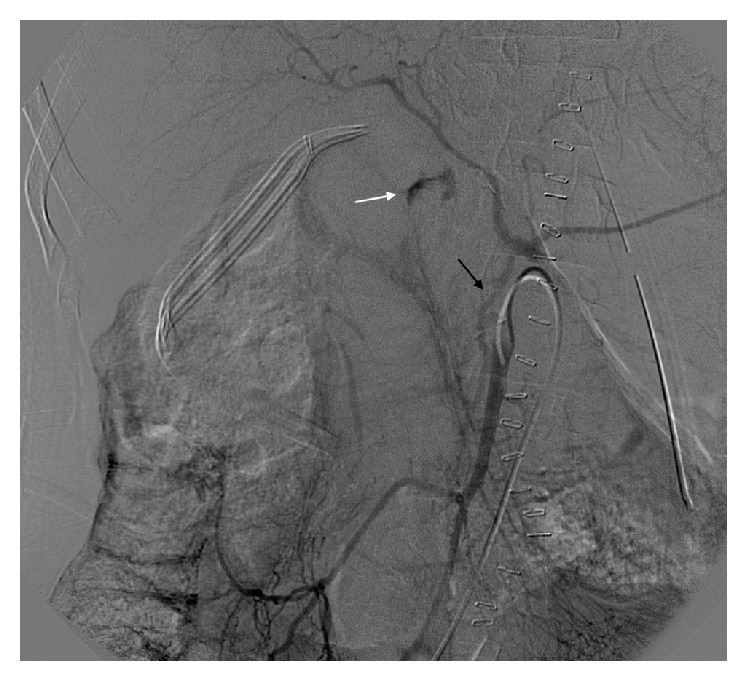
Selective angiography of superior mesenteric artery demonstrating an intraluminal blush in small bowel (white arrow). The Arc of Buhler clearly demonstrated (black arrow).

A nasogastric tube was placed, returning 700 cc of blood and the patient was taken to the interventional radiology suite to achieve hemostasis. Attempts to cannulate the celiac artery were unsuccessful given the high-grade celiac stenosis seen on the CT angiography. A lateral aortogram was performed which failed to identify the origin of the celiac artery. The superior mesenteric artery was then cannulated and angiogram demonstrated extravasation from a distal branch in the right upper quadrant (Figure 3).

**Figure 4:**
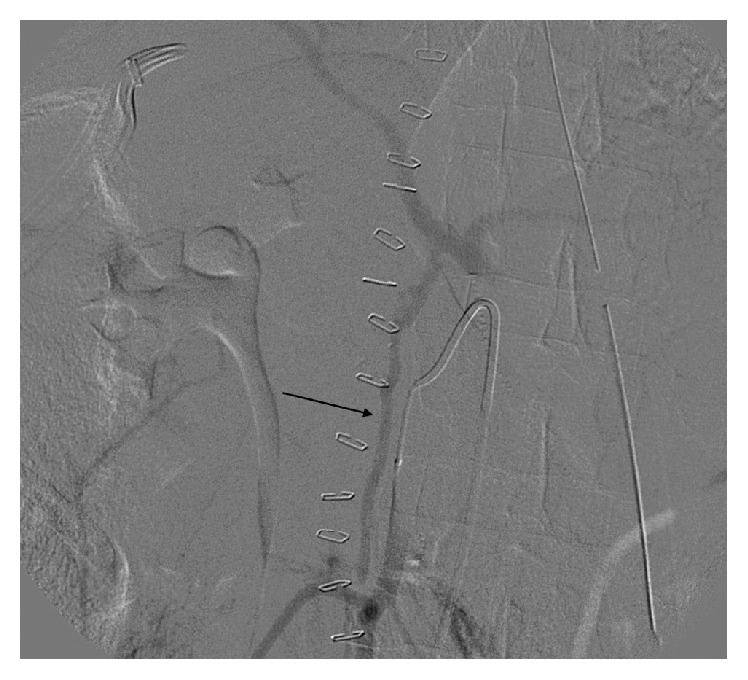
Selective angiography of superior mesenteric artery demonstrating the Arc of Buhler collateral between the SMA and celiac trunk (black arrow). Retrograde filling of celiac artery through Arc of Buhler, no filling of aorta indicating high-grade stenosis at the celiac origin.

Interestingly, contrast filling of the celiac artery was appreciated on this angiogram consistent with collateral circulation and communication between the superior mesenteric artery and celiac artery (Figures 3 and 4). Retrograde celiac filling also demonstrated high-grade stenosis at its origin. The bleeding had spontaneously stopped during the procedure.

Following IR intervention, the patient's hematocrit stabilized and slowly returned to normal limits. He experienced no further bleeding episodes. A follow-up CT showed concerns for a partial left hepatic lobe infarct. The remainder of his hospital course was complicated by bacteremia requiring antibiotic treatment. He was subsequently discharged on postoperative day 19.

## 3. Discussion

There are two main collaterals that occur between the celiac artery and the SMA: the gastroduodenal artery (GDA), a branch of the common hepatic artery, and the dorsal pancreatic artery, a branch of the splenic artery [10]. The AOB anomaly serves as a third route for collateral flow. The GDA supplies blood directly or indirectly to the stomach, duodenum, and head of the pancreas and retrograde flow would provide blood flow to the liver [11]. When considering a Whipple operation, it is crucial to account for any anatomical variants that may affect surgical planning. GDA ligation at its origin is an essential step during pancreaticoduodenectomy. With the proper ligation of this vessel, the arterial blood supply to the liver comes solely from the celiac artery via the common hepatic artery. In this case, the patient's blood supply to the liver would have been severely compromised given his high-grade celiac stenosis caused by MALS. He did have some liver sequelae as there was concern for a small segment of ischemia in the left hepatic lobe seen on postoperative CT. This was likely secondary due to a decrease in blood flow after GDA ligation during surgery. In the setting of celiac stenosis, Douard et al. describe clamping the GDA intraoperatively to determine if the blood flow to the hepatic artery is compromised by objective decrease in pulse of the artery. This can also help identify significant atherosclerotic disease or a replaced common hepatic artery, a much more common variant that affects the ability to perform a pancreaticoduodenectomy. Of note, this was done intraoperatively and a strong pulse was appreciated in the hepatic artery. The high-grade celiac stenosis in this case was rendered asymptomatic preoperatively secondary to the blood perfusion from collateral flow from the GDA and his persistent AOB which was supplying the celiac artery distal to the area of stenosis. In addition, secondary to the celiac occlusion the primary blood flow to the spleen was coming from the splenic artery that was being perfused by the AOB. Without the perfusion of this vascular anomaly, he would have faced devastating consequences.

Most reports in the literature describing the AOB are associated with finding a symptomatic aneurysm or describing the incidence of AOB after a retrospective review of angiographic films. There are reports describing celiac obstruction by an AOB aneurysm treated successfully with coil embolization [12, 13]. There have also been reports that describe an AOB with occlusion or high-grade stenosis of the celiac axis [11, 13–17]. AOB was found in one patient with SMA occlusion [11]. Only one other case report found MALS to be the culprit for celiac stenosis in the setting of an AOB [13] while others reported coincidence of celiac stenosis with development of aneurysms [1, 17–19].

There is one case in the literature of an incidental AOB with associated celiac occlusion in preoperative workup for a pancreaticoduodenectomy [16]. In this report, Wayne et al. found that the vascular anomaly had little to no effect on perioperative morbidity and outcomes, where we found significant morbidity. This could be secondary to the degree of celiac stenosis or size of the AOB. The incidence of the AOB is too low to determine if there is an actual relationship between the celiac stenosis and/or occlusion and persistent AOB although this relationship is reported in the literature. This phenomenon may be explained by dilation of a patent AOB for compensation purposes but this does not explain its embryologic persistence, as the celiac stenosis would have developed later in life.

This case underlines the importance of preoperative planning and image review prior to surgical intervention as it could drastically change the patient's outcomes. Unfortunately, it was only after retrospective analysis that the AOB and celiac stenosis were seen on preoperative imaging, albeit the findings were not as impressive as the postoperative and IR imaging. It is important to educate both radiologists and surgeons, on the possible anatomical variants that may be encountered in the vascular supply of the foregut.
